# Predictive modeling of antibiotic eradication therapy success for new-onset *Pseudomonas aeruginosa* pulmonary infections in children with cystic fibrosis

**DOI:** 10.1371/journal.pcbi.1011424

**Published:** 2023-09-06

**Authors:** Lucía Graña-Miraglia, Nadia Morales-Lizcano, Pauline W. Wang, David M. Hwang, Yvonne C. W. Yau, Valerie J. Waters, David S. Guttman

**Affiliations:** 1 Department of Cell and Systems Biology, University of Toronto, Toronto, Ontario, Canada; 2 Centre for the Analysis of Genome Evolution and Function, University of Toronto, Toronto, Ontario, Canada; 3 Department of Laboratory Medicine and Pathobiology, Toronto, Ontario, Canada; 4 Laboratory Medicine and Molecular Diagnostics, Sunnybrook Health Sciences Centre, Toronto, Ontario, Canada; 5 Department of Paediatric Laboratory Medicine, Division of Microbiology, The Hospital for Sick Children, Toronto, Ontario, Canada; 6 Department of Pediatrics, Division of Infectious Diseases, The Hospital for Sick Children, Toronto, Ontario, Canada; 7 Translational Medicine, Research Institute, Hospital for Sick Children, Toronto, Ontario, Canada; Burnet Institute, AUSTRALIA

## Abstract

Chronic *Pseudomonas aeruginosa* (Pa) lung infections are the leading cause of mortality among cystic fibrosis (CF) patients; therefore, the eradication of new-onset Pa lung infections is an important therapeutic goal that can have long-term health benefits. The use of early antibiotic eradication therapy (AET) has been shown to clear the majority of new-onset Pa infections, and it is hoped that identifying the underlying basis for AET failure will further improve treatment outcomes. Here we generated machine learning models to predict AET outcomes based on pathogen genomic data. We used a nested cross validation design, population structure control, and recursive feature selection to improve model performance and showed that incorporating population structure control was crucial for improving model interpretation and generalizability. Our best model, controlling for population structure and using only 30 recursively selected features, had an area under the curve of 0.87 for a holdout test dataset. The top-ranked features were generally associated with motility, adhesion, and biofilm formation.

## Author summary

Cystic fibrosis (CF) patients are susceptible to lung infections by the opportunistic bacterial pathogen *Pseudomonas aeruginosa* (Pa) leading to increased morbidity and earlier mortality. Consequently, doctors use antibiotic eradication therapy (AET) to clear these new-onset Pa infections, which is successful in 60%-90% of cases. The hope is that by identifying the factors that lead to AET failure, we will improve treatment outcomes and improve the lives of CF patients. In this study, we attempted to predict AET success or failure based on the genomic sequences of the infecting Pa strains. We used machine learning models to determine the role of Pa genetics and to identify genes associated with AET failure. We found that our best model could predict treatment outcome with an accuracy of 0.87, and that genes associated with chronic infection (e.g., bacterial motility, biofilm formation, antimicrobial resistance) were also associated with AET failure.

## Introduction

Cystic fibrosis (CF) is the most common fatal genetic disease among individuals of European descent. This disorder is caused by a dysfunction of the cystic fibrosis trans-membrane conductance regulator (CFTR) gene, for which over 2000 mutations have been identified [1]. The loss of CFTR function can cause a suite of systemic, physiological problems, although the greatest impact is in the lungs where abnormal thick mucus accumulation in the airways inhibits bacterial mucociliary clearance and allows microbial pathogens to thrive [2,3].

Bacterial airways infections in CF patients typically occur early on in life and can be difficult to treat. At an early stage of the disease, *Haemophilus influenzae* and *Staphylococcus aureus* typically colonize the lungs, but later *Pseudomonas aeruginosa* (Pa) becomes highly prevalent, chronically infecting with up to 32% of adults with CF [4–8]. Chronic infection with Pa is associated with decline in lung function, leading to increased morbidity and mortality [9]. Improved clinical care and treatment have increased the quality of life and life expectancy for CF patients considerably in the last 50 years [10]. In Canada, adult CF patients now represent more than 62% of all patients [11], yet bacterial infections, particularly with Pa, continue to pose a major threat. Consequently, the eradication of Pa infections early in life delays the establishment of chronic infections and improves long term lung function [12] and is therefore, crucial to enhancing the quality of life of CF patients [13].

Antibiotic eradication therapy (AET) is the standard procedure for treating new-onset Pa infections; although the protocol varies according to region and care facility, the success rate is relatively high; however, 20% to 40% of patients fail to clear the infection [14–16]. While there are likely many reasons for AET failure, the genetic makeup of the infecting Pa population is clearly an area of intense interest, and factors such as variation in the exopolysaccharide Psl have been shown to contribute to increased biofilm formation and tobramycin tolerance [17]. Despite this, there has not yet been a systematic study of the relationship between Pa genetic diversity and AET failure, or an attempt to predict the latter from the former.

Machine learning and related statistical genetic methods are now broadly used in microbiology to predict clinical outcomes, sources of infection, antibiotic resistance, and genetic variants underlying traits of interest [18–25]. While both supervised and unsupervised machine learning techniques have been useful in this context, there are several fundamental challenges to the application of machine learning in genomics. The first challenge is simply the scope of genomic diversity. Since each genetic variant (or input feature in the parlance of machine learning) is an independent variable, datasets inherently have very high dimensionality [26]. These features can be single nucleotide polymorphisms/variants (SNPs/SNVs), k-mers, unitigs (high-confidence, non-ambiguous contigs of assembled k-mers), or gene presence/absence. Dealing with high dimensionality data is non-trivial and if ignored will often lead to model overfitting. For this reason, it is often necessary to use feature selection or extraction techniques to avoid fitting random variation and non-informative features.

The second common problem in genomics is the lack of high-quality or high-confidence phenotypic data. This is particularly true for traits related to virulence, pathogenicity, and antibiotic susceptibility, since these traits are often measured in model or *in vitro* systems that differ from the ‘natural’ target system both biotically (e.g., different host or microbial interactions) and abiotically (different environment and growth conditions).

Other challenges to the application of machine learning to genomics include predicting complex traits, whether these complexities are brought about through polygenic inheritance, epistasis, gene-by-environment interactions, variable penetrance, variable expressivity, genetic heterogeneity (i.e., genocopies), or phenocopies [27–30]. In addition, sampling biases, and non-independent evolutionary histories (i.e., population structure) among samples can result in hidden and complex covariation among input features [25,31–36]. Despite these challenges, machine learning and other statistical genomic approaches have proven to be extremely valuable tools for dissecting complex genomic data [23–25,37].

The aim of this work was to build a machine learning model to predict new-onset AET success or failure using whole genome sequence from Pa isolates cultured from new-onset infections in CF patients (prior to antibiotic treatment). We performed Random Forest and Extreme Gradient Boosting predictive modeling, both consist of an ensemble of decision trees, each providing an outcome based on different subsets of variable genomic unitigs. Random Forest modeling has become increasingly popular in genomics, because it has shown high performance with small sample sizes, high-dimensional data, and complex data structures [38–40]. Additionally, decision tree-based models allow input variables to be ordered according to their importance, which enabled us to identify which genomic variants have a strong influence on the outcome [41]. We used a nested cross validation (NCV) design [42], population structure control to control for non-independent evolution histories of the Pa strains, and feature selection to reduce the size of the input feature set and identify those variants that may be associated with AET failure [38,43].

## Results

### Genomic and phenotypic diversity of Pa isolates

**Fig 1:**
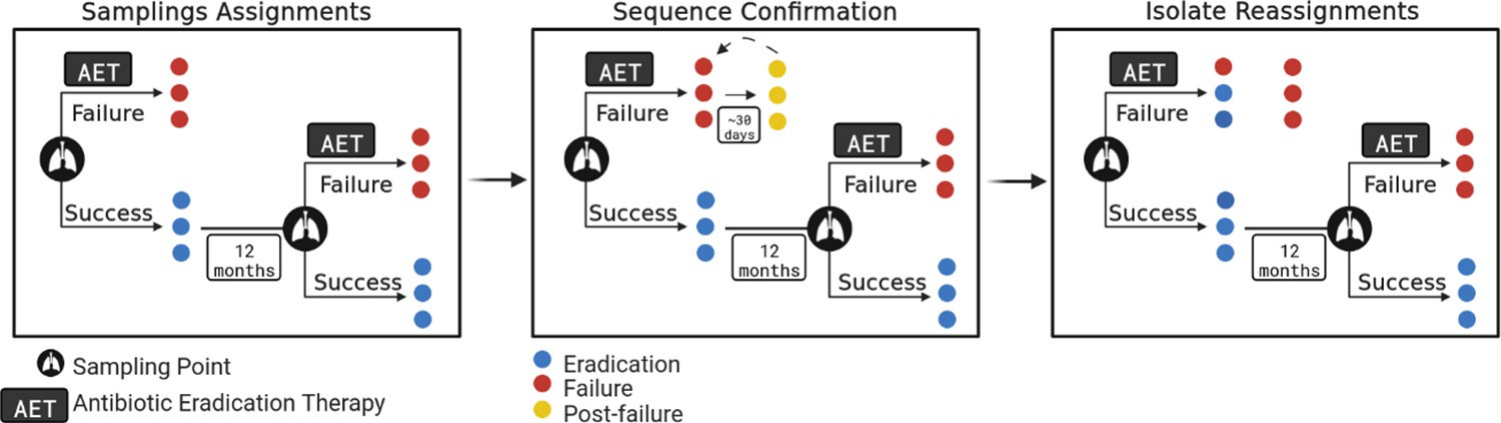
Overview of sampling strategy. Sampling scheme for new-onset *P*. *aeruginosa* (Pa) infections and antibiotic eradication therapy (AET) with paths for treatment success or failure. New-onset Pa infections (identified by the lung icon) were defined as the first lifetime acquired infection or having a Pa-positive sputum culture after having at least three Pa-negative sputum cultures within the 12 months prior. More than one isolate was collected if morphotype diversity was observed. AET (inhaled tobramycin multi-step protocol) either led to successful eradication (blue) or failure to eradicate (red). Sequence confirmation and isolate reassignment paths were performed for patients with high genomic diversity AET failure infections. Persistent isolates (yellow) were collected, sequenced, and compared to pre-AET isolates to identify which clones survived AET. If a post-failure isolate was found to be nearly identical to a failure isolate, then that failure isolate would retain its failure label. If the post-failure isolate was not closely related to a prior failure isolate, then those prior isolates were reassigned to the eradication success category.

Bacterial isolates were retrieved from a cross-sectional study of CF pediatric patients (aged 0–18 years, mean = 9.7, sd = 3.5) with new-onset Pa infections during the period 2011–2016 at the Hospital for Sick Children, Toronto, Canada [44]. A total of 440 Pa isolates were recovered from sputum collected from 70 patients prior to an inhaled tobramycin multi-step protocol hereafter referred to antibiotic eradication therapy (AET) [15]. To assess within-patient diversity, one or more isolates were sequenced for each patient, depending on the number of colony morphologies recovered from the sputum sample. Sample isolates were labeled as eradication if the Pa infection was successfully cleared by AET or failure if AET was unsuccessful (Fig 1).

New-onset infections usually refer to those acquired for the first time in the patient’s life. In this study, we also considered new-onset infections to be those acquired for the second or third time, but with at least 12 months between infections. Among the 70 patients, five patients had a first infection that was cleared followed by a second infection that failed AET, and 12 patients were infected more than once with successful AET each time. In total, there were 90 infection episodes among the 70 patients, of these, 67 (74.4%) were eradicated and 23 (25.6%) failed eradication. Of the 440 sequenced Pa isolates, 124 (27.2%) were recovered prior to AET failure and 316 (72.8%) were recovered prior to AET success.

**Fig 2:**
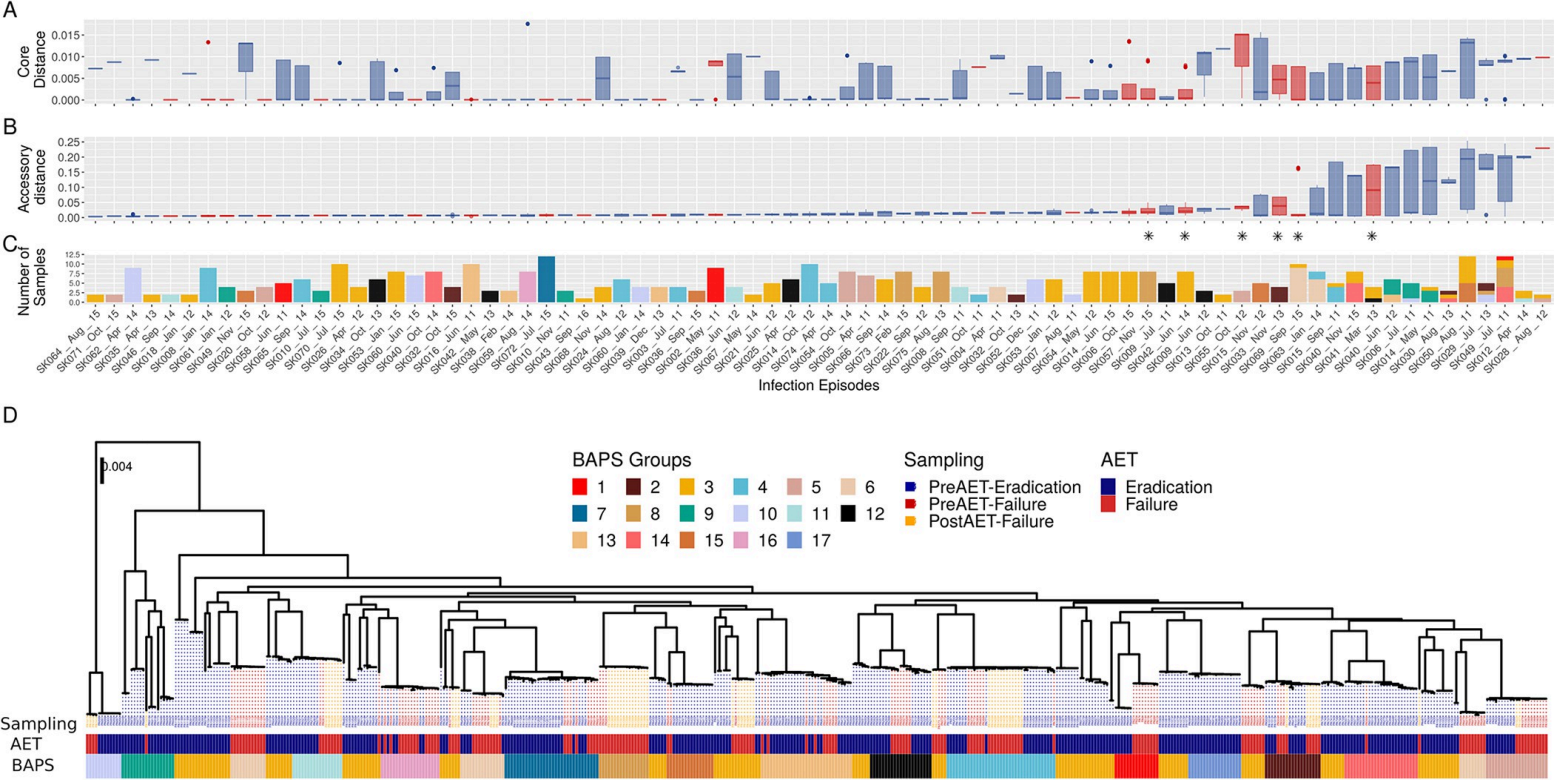
Genomic Diversity. (A) Core genome distance within each infection episode as estimated by MASH [95]. (B) Accessory genome Bray-Curtis distance within each infection episode estimated from ROARY analysis of gene presence/absence [92]. The infection episodes in both panels (A) and (B) are displayed in ascending order according to accessory genome distance median. (C) Number of isolates recovered in each infection episode and the proportion of those that correspond to each BAPS group. (D) Core genome, midpoint-rooted phylogeny of the evolutionary relationships between the 494 Pa strains. Sampling point is represented by the color coding of the dashed lines leading from the terminal nodes to the metadata rows, with blue showing pre-AET success (i.e., eradication), red showing pre-AET failure, and orange showing a post-failure isolate. The first annotation bar below the tree indicates AET outcome, while the second indicates the BAPS group.

We performed pangenome analysis to evaluate sample diversity within infections. While core genome distance between the samples was low (Fig 2A), we found high levels of accessory genome variation among 15 eradication and seven failure infection episodes (Fig 2B). Within-infection diversity could affect the AET outcome classification (i.e., eradication vs. failure) of individual isolates since the outcomes were assigned at the patient level. While this was not a problem when the infection was successfully cleared, it was a concern in AET failure patients since the cause of treatment failure could have been driven by only one of the multiple isolates recovered from that patient at that sampling time, while other pre-AET strains could have been sensitive to treatment and culled from the population. To confirm the AET outcome for each strain, we sequenced 55 isolates obtained after AET failure from ten patients (‘post-failure’ isolates, Fig 1). Unfortunately, only six of these ten patients had accessory genome diversity (Fig 2B, asterisk). There were an additional two patients (SK006 and SK028) that showed high pre-failure accessory genome diversity for which we could not obtain post-failure isolates The post-failure isolates are identified with yellow labels in Figs 1B and 2D.

We found that most post-failure genomes shared a recent common ancestor with at least one pre-AET failure genome from the same patient (S1 Fig). We also evaluated the accessory genome distance between pre-failure and post-failure isolates for four patients with pre-failure diversity to determine if any of the pre-failure strains should be individually reassigned to the eradication phenotype. We reasoned that any pre-failure strain that was sensitive to AET would not be present in the post-failure collection. Ultimately, out of 28 pre-AET failure isolates, only three isolates from three patients were markedly different from the post-treatment collection, and therefore, were reassigned from a failure to eradication phenotype (S1 Fig). Given that there was very little genetic distance between non-reassigned pre-failure and post-failure genomes at both core and accessory genomes, and that sampling time after treatment was only approximately 30 days, we decided to include the post-failure isolates in the machine learning analysis (Fig 1). This had the benefit of increasing our sample size and ameliorated the difference between the number of eradicated and failed isolates (S1 Fig). The final sample set was therefore of 494 isolates, with 177 corresponding to AET failure samples (S1 Table).

Pa isolates in new-onset infections are usually acquired from the environment or upper-airway [45–48], therefore we expected high genetic diversity within the sample. To explore this, we built a core genome phylogeny including three reference genomes (PAO1, PA14, PA7) that are found in the three major Pa lineages previously identified by Ozer and colleagues [49]. We found that most of our isolates clustered in lineage 1, represented by the PAO1 reference genome, while a small number clustered in lineage 2, represented by the PA14 reference genome. None of our isolates clustered in lineage 3, represented by the PA7 reference genome (S1 Fig).

We observed that the AET success/failure outcome was highly correlated with the phylogenetic structure of the sample collection (Fig 2D), with most of the clades containing isolates assigned to one of the two AET outcome phenotypes. This correlation between phenotypes of interest and the phylogenetic structure impacts machine learning predictions since it can introduce spurious associations between genomic diversity and the AET success/failure outcomes. This population structure bias, also known as population stratification or lineage effects, is caused by non-independent (i.e., correlated) evolutionary histories among strains in the sample [26]. Such biases have been extensively discussed in the context of both genome-wide association studies and machine learning predictive modeling [25,31–34,50–52].

We assessed and controlled for population structure using Bayesian Analysis of Population Structure (BAPS) [53–55]. The analysis supported the existence of 17 BAPS subpopulations or groups (Fig 2C and 2D). Only two BAPS groups (14 and 17) were composed exclusively of AET success isolates, while the rest had representatives of both outcomes in different proportions. Most of the infection episodes were caused by homogeneous groups of isolates corresponding to a single BAPS subpopulation, with 76 of the 90 (84.4%) infection episodes caused by a single closely related clone (Fig 2C and 2D). Counterintuitively, BAPS group 3 includes multiple distinct clades that span the entire tree. This classification reflects the genetic background of the strain collection and includes those clades that do not cluster into smaller distinct cluster, perhaps due to recombination (Dr. Gerry Tonkin-Hill personal communication) [56]. Of seven AET failures with high accessory genome variation, three were polyclonal, while of the 15 eradication episodes with high accessory genome variation, 11 were polyclonal (Fig 2C). We also observed multiple closely related clades that included strains obtained from multiple patients, indicating that person-person transmission could be of importance [57–59].

**Fig 3:**
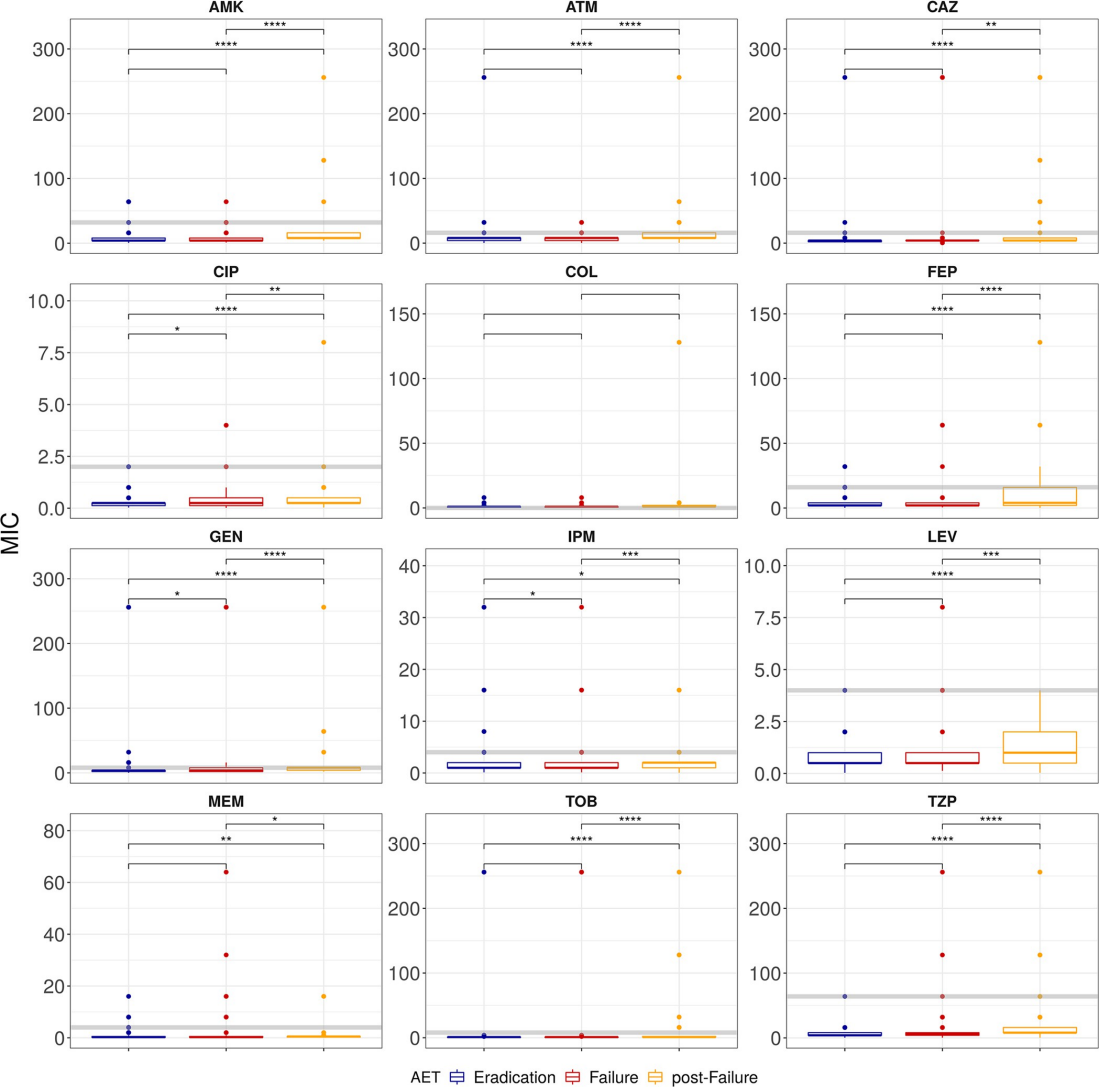
Antimicrobial susceptibility tests. Minimum inhibitory concentrations (MIC in ug/ml) levels of 12 antibiotics, for AET success (i.e., eradicated), AET failure, and post-AET failure samples. The grey horizontal line indicates the breakpoint value. AMK: Amikacin, ATM: Aztreonam, CAZ: Ceftazidime, CIP: Ciprofloxacin, COL: Colistin, FEP: Cefepime, GEN: Gentamicin, IPM: Imipenem, LEV: Levofloxacin, MEM: Meropenem, TOB: Tobramycin, TZP: Piperacillin \+ Tazobactam. Statical significance is represented with asterisks: ns: p>0.05, *: p< = 0.05; **: p< = 0.01; ***: p< = 0.001.

Antimicrobial susceptibility testing (AST) was performed for 12 antibiotics for all the isolates via broth microdilution assays. We observed significant differences in the minimum inhibitory concentrations (MICs) between failed and eradicated isolates for ciprofloxacin (CIP, Chi square, p = 0.003), gentamicin (GEN, p = 0.002) and imipenem (IMP, p = 0.03) (Fig 3). Inhaled tobramycin treatment can achieve very high airways concentrations, therefore is the standard treatment applied to patients regardless of the tobramycin MIC value obtained with traditional AST. Despite this, no association was found between tobramycin resistance and AET success/failure (Chi square, p value = 0.09). Post-treatment isolates were also tested for antimicrobial susceptibility and found to have increased MIC levels for most of the 12 antibiotics tested (Fig 3), revealing a rapid change in the antibiotic resistant ability of the Pa population during treatment of the infection. Only 13 isolates showed high levels of tobramycin resistance (MIC >256 μg/mL), with eight (61.5%) occurring in strains from AET failure samples and five (38.5%) from AET success sample. Resistance to tobramycin with MIC ≥ 16 μg/mL, was found in 6.8% of the cases of AET failure and 1.6% of the AET success cases.

### Genome-based predictive modeling of AET success/failure outcomes

#### Input features

Input features representing the genetic variation of the sample population must be extracted from whole genome sequence data to build ML predictors. While most statistical genomic applications use SNPs, SNVs, or gene presence/absence data [19,21,60–62], short sequences such as k-mers or unitigs are becoming increasingly popular since they inherently incorporate polymorphisms, insertion/deletions, and gene presence/absence [63,64]. Here we used a presence/absence matrix of unitigs spanning the pangenome as input features to predict AET success/failure. Unitigs are high confidence contigs (i.e., assembled sequence reads) that have no mismatches or ambiguous residues. In addition to being of high confidence, unitigs also have the advantage of being less redundant than k-mers [63]. In order to reduce the number of input features, we selected a non-redundant set of unitigs by using only one representative unitig from a set of identical patterns of presence/absence among the strain collection. We also removed unitigs with frequencies less than 5% and greater than 90% (reducing the number of features from 542,296 to 425,005) since these are highly unlikely to be strongly associated with our AET success/failure outcome, which was observed in 36% of the samples.

#### Machine learning predictor design

**Fig 4:**
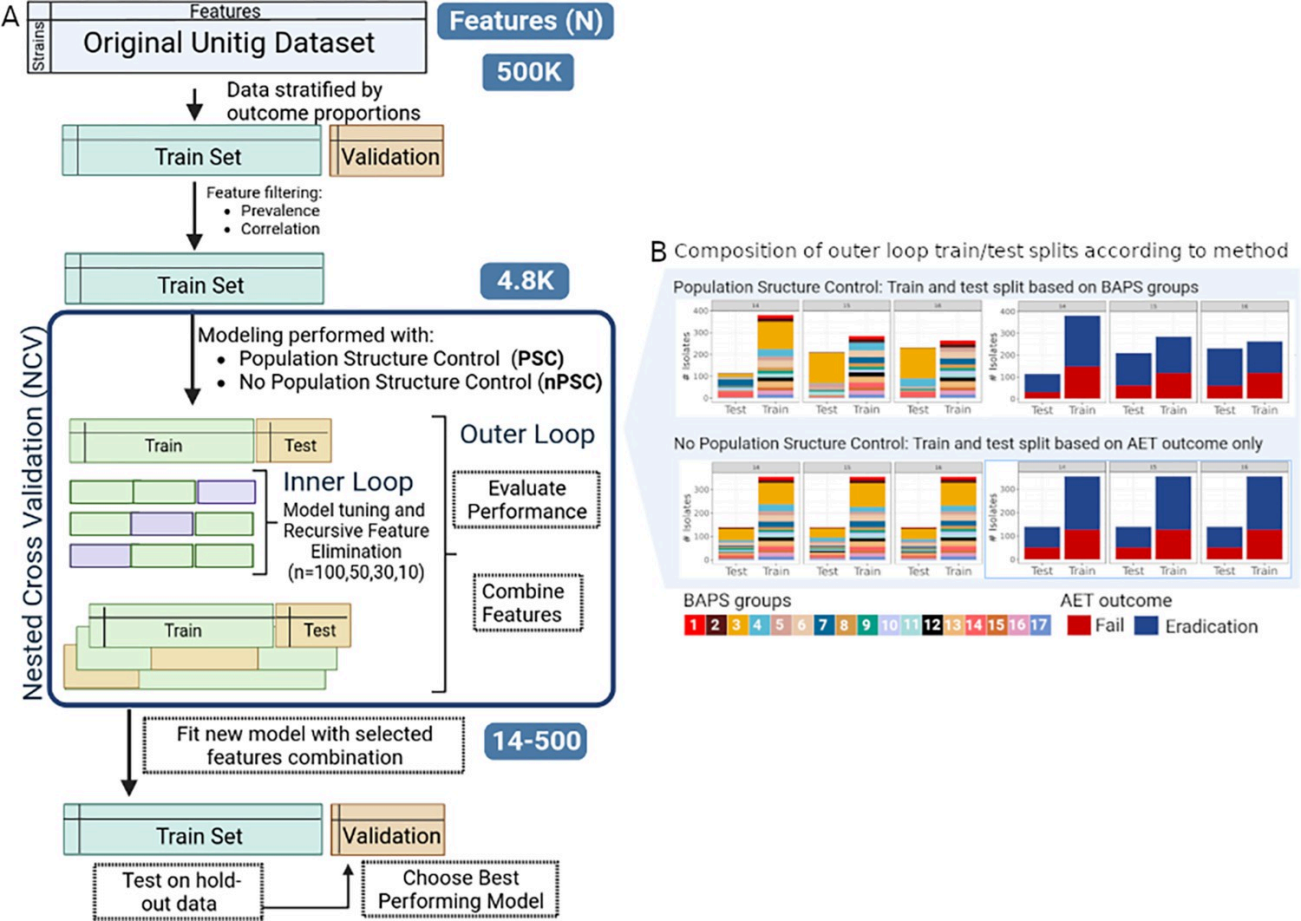
Machine learning overview. (A) Workflow for the prediction of AET outcome based on genomic unitig diversity. The one-hot-encoded unitig data was divided into a training and a validation set stratified by the outcome proportions. Feature correlation filters were applied on training data to remove redundant features, thereby reducing the feature dimension from >500k to 4800. We used recursive feature elimination (RFE) in the nested cross validation (NCV) loops to further reduce the number of features in a model dependent manner. Separate modeling was done with no population structure control (nPSC) and population structure control (PSC), which was implemented by blocking the data based on BAPS groups. The feature combinations obtained during NCV were used to fit new predictors on whole training data. Finally, the model was evaluated on the hold-out validation set. The same validation set was used to evaluate the performance of all models. (B) Three different examples to illustrate how samples are split during the NCV’s outer split into training and test, depending on the method used.

We initially divided the dataset into a train set composed of 90% of the samples, and a validation set composed of 10% of the samples. This split was done while maintaining AET outcome proportions, and the same validation set was used to evaluate the performance of all models. We used a nested cross validation (NCV) design to optimize our model, train/test blocking by BAPS groups for population structure control (PSC), and recursive feature elimination (RFE) with random forest for feature selection (Fig 4A). NCV has a double loop analytical structure, with an inner loop used for model/parameter selection, and an independent outer loop that assesses model quality. This approach maximized the use of our small sample size, and enabled feature selection and hyperparameter tuning in a way that minimized the likelihood of model overfitting [42,65–67]. Given the imbalanced nature of the data we used area under the receiver operating characteristic (ROC) curve (AUC) as the performance measure. Train and test AUC can be compared to evaluate over-fitting and population structure impact. We also used precision, recall, and the F1 score for the positive (AET failure) and negative (AET eradication) classes to assess model performance. Having a high recall of the positive class is extremely important because it reflects a low number of false negatives or a small type II error, which we are particularly interested in reducing (i.e., reduce the number of false eradication predictions) (S3 Fig).

Since models built using an excess of features relative to the number of samples can suffer from overfitting and achieve poor generalization [25,43], we performed feature reduction through a combination of filters and wrappers methods. We used a chi-squared association test to remove features with no association with the AET outcome and identified and removed highly correlated features (Pearson correlation coefficient > 0.7) in the training data set. This reduced the number of unitig patterns from 425,005 to 4800.

We used RFE for model-dependent feature selection [68]. RFE is a wrapper-type feature selection algorithm that ranks features by importance and recursively removes the least important ones until the desired number of features remains. During the NCV inner loop, hyperparameters are optimized and the best model obtained is then used with RFE during the outer loop. We ran four independent pipelines selecting for different numbers of features during the RFE step (n features = 10, 30, 50, and 100) to account for the random nature of the algorithm and the impact of multiple feature combinations on the performance of our predictor [68]. The features selected with RFE in each NCV iteration outer loop were combined to fit a new predictor on the original training set and the performance tested on the hold-out validation set.

Finally, we used PSC to account for correlated or non-independent evolutionary histories among our strains during the NCV step. PSC was implemented using BAPS group blocking when splitting the NCV outer loop train and test sets. In other words, we assigned each BAPS group (i.e., all of the strains assigned to a particular BAPS group) to either the train set or the test set, while maintaining class proportions (Figs 4B and S2). The goal of blocking via BAPS groups was to impose a restriction to the learning process, making the model train only on specific subpopulations and test on others. This allowed us to assess the influence of genomic data structure on performance and determine how well the model would perform on strains from clades that were not used for model training. Controlling for population structure is critically important for the development of generalizable models that make robust predictions for all strains, regardless of their evolutionary histories. This approach is especially important for bacterial species with relatively low recombination rates such as Pa. We assessed the impact of PSC by comparing the performance of models with PSC to those with no population structure control (nPSC), which maintained AET outcome proportions for the outer NCV split but did not take into consideration any population structure (Figs 4B and S2).

**Fig 5:**
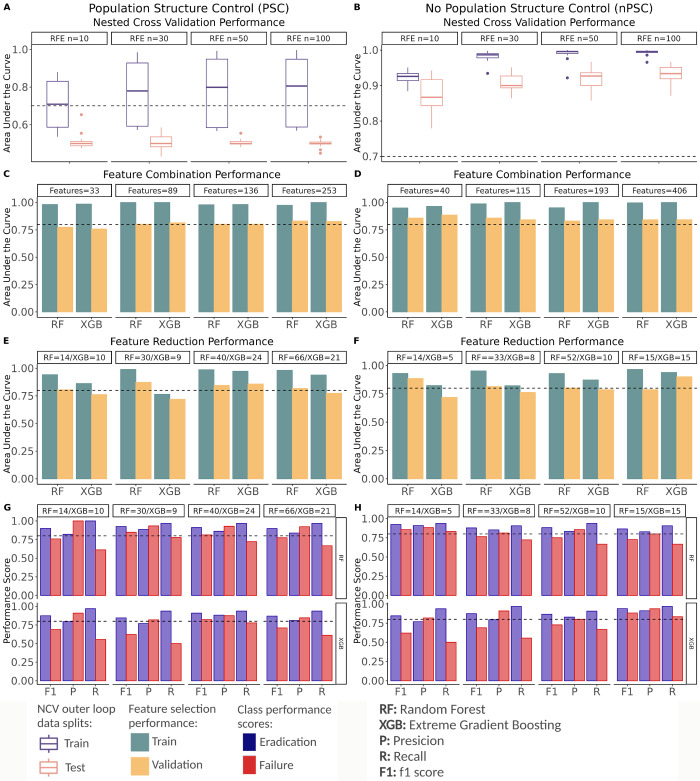
Model performance using recursive feature elimination (RFE). (A) Area under the curve (AUC) values for the training and test dataset obtained during the PSC NCV analysis with different numbers of features selected (i.e., 10, 30, 50, and 100) during the RFE step. (B) The same results with nPSC. Note that the y-axes are on different scales. The dashed line at 0.7 indicates the threshold for feature selection. The size of the feature combination set for each independent run is shown in the box on top. (C) Train and validation AUC obtained using the reduced set of features previously selected with PSC NCV. (D) Train and validation AUC obtained using the reduced set of features previously selected with nPSC NCV. (E-F) Train and validation AUC obtained after feature size reduction based on the training feature importance. (G-H) Precision (P), recall (R) and F1 score (F1) for the AET outcomes (blue indicating AET success, i.e., eradication, and red indicating AET failure) obtained using a further feature size reduction based on the training feature importance. The dashed line at 0.8 provides a common reference. The best performing models are indicated with a black square.

The effect of including PSC was substantial (Fig 5A). Model performance varied widely between outer loop iterations. Notably for some of the splits we obtained very poor training performance (mean train AUC = 0.75, sd = 0.16). Furthermore, testing performance was very low (AUC < 0.7), which means that even when the learning process showed high performance, the generalization power to other BAPS groups was low (mean test AUC = 0.51, sd = 0.03). This behavior was maintained despite the number of features selected in independent RFE runs. In contrast, when PSC is not applied during NCV, model performance was consistently high across outer splits (mean train AUC = 0.97, sd = 0.03), both for training and testing (mean test AUC = 0.91, sd = 0.04) (Fig 5B). Still, train/test AUC differences indicated that the model was overfitting when the number of features selected during RFE was set higher than 10 or 30 (Fig 5A and 5B).

We ran four independent NCV iterations that differed in the numbers of retained features and combined all the features within each of the iterations. This was done for both the PSC and nPSC pipelines; thereby, generating eight feature sets ([10, 30, 50, or 100 retained features] by [PSC or nPSC]) (Fig 5). The goal of this feature combination step was to obtain features that could provide accurate predictors independently of the train/test split in order to reduce biases that plague models with small, unbalanced, and highly correlated datasets.

The eight feature sets were used to train new predictors with the entire training set, and model performance was evaluated on the hold-out validation data (Fig 5C and 5D). At this point we sought to evaluate the performance of different features sets, so the main difference between the models was in the features set identity not in the train/test split, which is the same in every case.

We used two decision tree-based algorithms, Random Forest (RF) and Extreme Gradient Boosting (XGB) for model construction and found that models trained with feature combinations achieved high performance irrespective of whether PSC was used during the NCV process (Train AUC > 0.8, Validation AUC > 0.75) (Table 1). Unfortunately, there were signs of overfitting (i.e., the performance of the training set is significantly better than the test set), particularly when using large feature combination sets (Fig 5C and 5D). Table 1 provides detailed performance scores for each step.

**Table 1:** Unitig Model Performance.

**Population Structure Control (PSC) Models**				
**Nested Cross Validation (NCV)**				
N Features	100	50	30	10
Train AUC^1^ (sd)	0.77 (0.18)	0.77 (0.18)	0.76 (0.17)	0.70 (0.13)
Test AUC (sd)	0.50 (0.02)	0.50 (0.02)	0.51 (0.04)	0.51 (0.0.4)
**Random Forest (RF) Feature Combination**				
N Features	253	136	89	33
Train AUC	0.97	1.00	0.98	0.98
Test AUC	0.82	0.80	0.80	0.77
**Random Forest (RF) Feature Reduction based on Gini Importance**				
N Features	66	40	30	14
Train AUC	0.98	0.99	0.99	0.94
Test AUC	0.82	0.87	0.85	0.81
**Extreme Gradient Boosting (XGB) Feature Combination**				
N Features	253	136	89	33
Train AUC	1	0.98	1	0.99
Test AUC	0.83	0.80	0.81	0.76
**Extreme Gradient Boosting (XGB) Feature Reduction via Feature Importance**				
N Features	21	24	9	10
Train AUC	0.94	0.97	0.76	0.86
Test AUC	0.77	0.86	0.71	0.76
**No Population Structure Control (nPSC) Models**				
**Nested Cross Validation (NCV)**				
N Features	100	50	30	10
Train AUC (sd)	0.99 (0.01)	0.99 (0.02)	0.98 (0.02)	0.92 (0.02)
Test AUC (sd)	0.93 (0.02)	0.92 (0.03)	0.92 (0.02)	0.87(0.05)
**Random Forest (RF) Feature Combination**				
N Features	406	193	115	40
Train AUC	1.00	0.95	0.99	0.95
Test AUC	0.84	0.83	0.86	0.86
**Random Forest (RF) Feature Reduction based on Gini Importance**				
N Features	78	52	33	14
Train AUC	0.97	0.93	0.95	0.93
Test AUC	0.79	0.80	0.81	0.89
**Extreme Gradient Boosting (XGB) Feature Combination**				
N Features	406	193	115	40
Train AUC	1	1	1	0.96
Test AUC	0.84	0.84	0.84	0.89
**Extreme Gradient Boosting (XGB) Feature Reduction via Feature Importance**				
N Features	15	10	8	5
Train AUC	0.94	0.87	0.82	0.82
Test AUC	0.90	0.79	0.76	0.71

1 AUC, area under the curve

**Table 2:** Unitig Classification Report.

**Population Structure Control (PSC) Models**								
**Random Forest (RF)**								
N Features	66		40		30		14	
AET ^1^	Success	Failure	Success	Failure	Success	Failure	Success	Failure
Precision	0.84	0.92	0.86	0.93	0.88	0.93	0.82	1.00
Recall	0.97	0.67	0.97	0.72	0.97	0.77	1.00	0.61
F1 Score	0.90	0.77	0.91	0.81	0.93	0.85	0.90	0.76
**Extreme Gradient Boosting (XGB)**								
N Features	21		24		9		10	
AET ^1^	Success	Failure	Success	Failure	Success	Failure	Success	Failure
Precision	0.81	0.85	0.88	0.88	0.77	0.82	0.79	0.91
Recall	0.94	0.61	0.94	0.78	0.94	0.50	0.97	0.56
F1 Score	0.86	0.71	0.91	0.82	0.85	0.62	0.87	0.69
**No Population Structure Control (nPSC) Models**								
**Random Forest (RF)**								
N Features	78		52		33		14	
AET ^1^	Success	Failure	Success	Failure	Success	Failure	Success	Failure
Precision	0.83	0.80	0.83	0.86	0.86	0.81	0.90	0.88
Recall	0.90	0.67	0.94	0.67	0.91	0.72	0.94	0.83
F1 Score	0.87	0.72	0.88	0.75	0.88	0.76	0.92	0.86
**Extreme Gradient Boosting (XGB)**								
N Features	15		10		8		5	
AET ^1^	Success	Failure	Success	Failure	Success	Failure	Success	Failure
Precision	0.91	0.94	0.83	0.8	0.79	0.91	0.77	0.82
Recall	0.97	0.83	0.90	0.67	0.97	0.56	0.94	0.50
F1 Score	0.94	0.88	0.87	0.73	0.87	0.69	0.85	0.62

^1^ AET, antibiotic eradication therapy

#### Feature selection using feature importance

To further refine our models, we eliminated features with limited predictive power based on their importance calculated during training [69]. In the case of RF, we used Gini Importance, also known as Mean Decrease Impurity, which is a measure of how well a feature splits samples with different outcomes. For XGB, we used the built in feature importance measure, calculated as the total reduction of the logloss, which is a measure of classification error. The reduced feature sets were then used in a subsequent round of predictor training. In some cases, the removal of poorly informative features did not have a major impact on performance, while in other cases it helped reduce the difference between train and test AUC (reducing overfitting) (Fig 5E and 5F and Table 2). Based on train/validation performance differences and the recall and precision scores for both classes (Fig 5G and 5H), the best performance with RF was achieved with 30 features obtained with the PSC pipeline (Train AUC = 0.99, Test AUC = 0.87, Precision: failure = 0.93, eradication = 0.88, Recall: failure = 0.78, eradication = 0.96), and a combination of 14 features obtained with nPSC pipeline (Train AUC = 0.93, Test AUC = 0.88, Precision: failure = 0.88, eradication = 0.91, Recall: failure = 0.83, eradication = 0.94) (Fig 5E and 5G). When using XGB, the best performance was achieved with 24 features obtained with the PSC pipeline (Train AUC = 0.97, Test AUC = 0.86, Precision: failure = 0.88, eradication = 0.88, Recall: failure = 0.78, eradication = 0.93), and 15 features obtained with the nPSC pipeline (Train AUC = 0.96, Test AUC = 0.83, Precision: failure = 0.87, eradication = 0.86, Recall: failure = 0.72, eradication = 0.93) (Fig 5F and 5H). Detailed performance scores for all fitted models are in Table 2.

**Fig 6:**
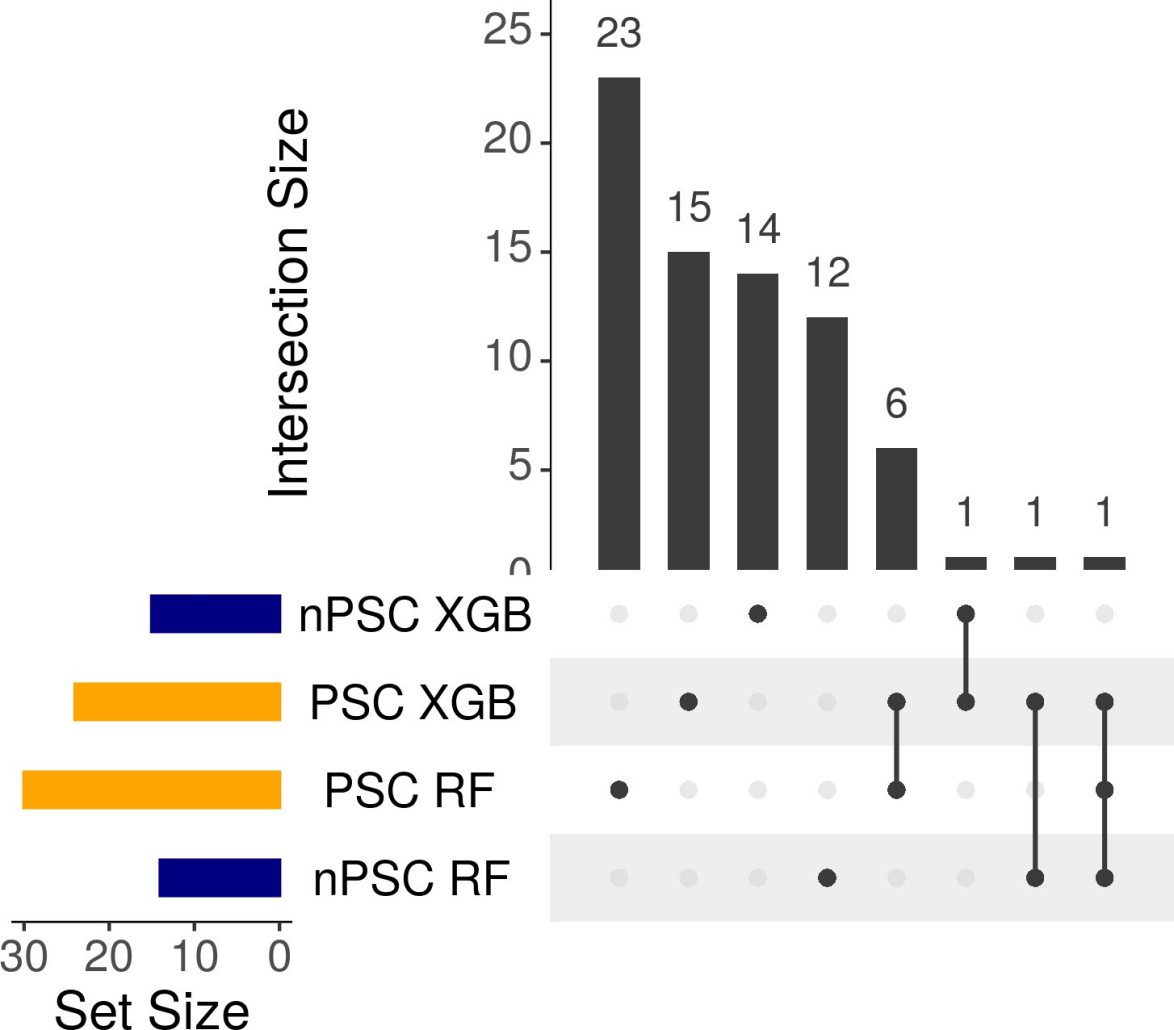
Feature overlap between the best performing models. The intersection plot shows the overlap between the selected feature sets. Sizes are displayed as horizontal bars on the lower left corner of the image, in blue RF and XGB selected features with the nPSC pipeline, in orange. RF and XGB instances of the PSC pipeline. Intersection sizes are shown as individual vertical bars. Independent pipelines involved in each intersection are identified with connected black circles under the vertical bars. Unconnected circles represent features that are found exclusively in the corresponding set.

Although similar performance can be achieved with and without the inclusion of PSC, the features selected with the two approaches differed (Fig 6). Considering the features selected in the best performing models, seven features are shared between PSC models, only three selected in both PSC and nPSC final models, and there is one feature that was consistently selected with PSC XGB and RF, and nPSC RF.

#### Testing the predictive power of randomly selected features

Given that similar performance can be achieved with different sets of features, we compared the performance of our best model to 500 models trained on 25 random features selected from the 4800 non-correlated input variables (S4 Fig). We found that most random groups of features lead to lower performance than our pipelines, although some combinations provide very good performance that even outperformed our more rigorous selection procedure discussed above. The test AUC from our best PSC and nPSC models fell at the 75th percentile of the random feature distribution. The high performance of some of the random feature models is likely due to many of features being strongly correlated with the phylogeny; thereby, supporting the use of PSC to reduce lineage effects.

#### Assessing the impact of population structure control (PSC)

**Fig 7:**
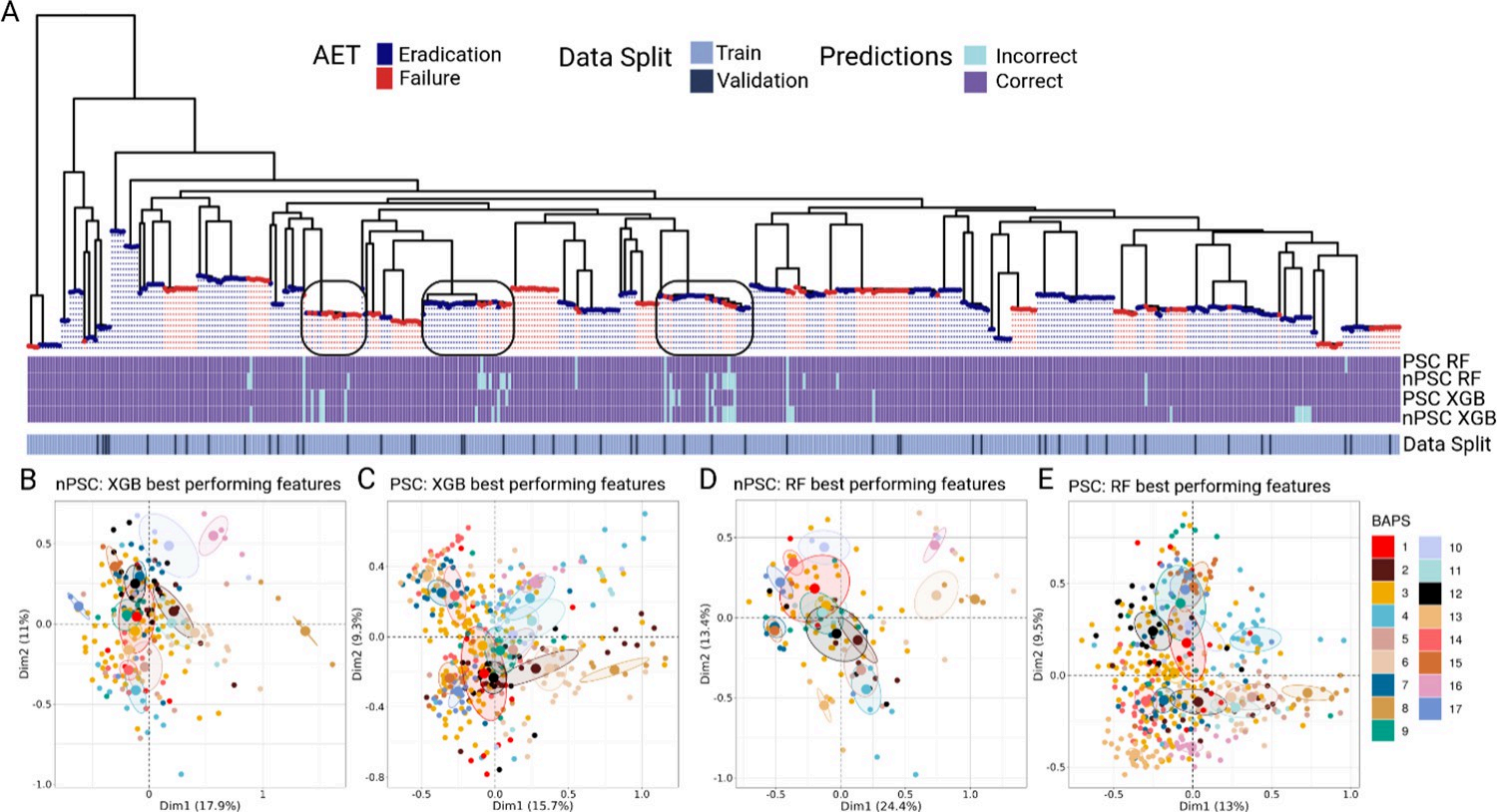
Assessing the impact of population structure control. (A) Core genome phylogeny with tips colored according to AET outcome. The annotation rows correspond to predictions obtained with the best performing models of the PSC and nPSC pipelines (purple = correct prediction, blue = incorrect prediction). The last row (Data Split) indicates whether the samples were part of the training or hold-out validation sets. (B-E) Multiple Correspondence Analysis (MCA) plots showing the clustering of the 494 samples based on the presence/absence pattern of the best performing features (i.e., unitigs) obtained with nPSC XGB (B), PSC XGB (C), nPSC RF (D), PSC RF (E). Colors indicate BAPS groups.

To determine if PSC could reduce lineage effects, we mapped the correctly and incorrectly predicted samples from the best-performing models onto the phylogeny (Fig 7A). Phylogenetic mapping showed that the nPSC pipeline errors were primarily concentrated in clades with closely related strains that had different AET outcomes, whereas PSC is able to correctly predict these cases (Fig 7A black squares). Since the AET outcome is highly correlated with the phylogeny, the model can learn from the phylogenetic signal and still achieve high performance, this behavior has been observed before [18]. The highlighted clades in Fig 7A contain isolates with different AET outcomes that can be accurately predicted with RF PSC pipeline, but not with the nPSC pipeline. This is an important finding since it indicates that features selected with PSC contain information that is independent of the phylogeny and thus should provide higher generalization power. Nevertheless, we cannot assign any causal relationship between the features we selected and the AET outcome unless we can distinguish between features that predict kinship and features that predict AET independently of phylogeny.

We used multiple correspondence analysis (MCA) to evaluate how well the feature combination from the best performing models, selected by the PSC and nPSC pipelines, delineate the BAPS groups (Fig 7B and 7E). This analysis found that BAPS groups were more intermixed when PSC was used for both RF an XGB approach, as can be seen by the overlapping confidence ellipses and more clustered centroids on the MCA plots (Fig 7C and 7E). This indicates that the PSC pipeline did a better job in creating a feature dataset that was population structure independent, and therefore, was better suited for finding causal variants underlying AET failure irrespective of the clonal background.

The finding that individual unitig patterns showed low correlation values with the AET outcome both in the PSC and nPSC groups of features (max absolute values 0.36 and 0.4 respectively) indicates that there may be multiple evolutionary routes to persistence in the lung during early stages of the infection. Furthermore, some of those features were clearly associated with persistent samples, with most of the isolates in that group sharing the same pattern (S5 Fig). This is likely due to differential fitness and survival of certain variants after treatment failure, which is consistent with our earlier finding that persistent isolates had increased antibiotic tolerance compared to the isolates obtained prior to AET.

### Genes associated with AET success/failure outcomes

We refined our feature set to focus annotation efforts on those features most strongly associated with the AET outcome during model validation. This step also has the added advantage of reducing model overfitting since we did not want to annotate features that were only useful for training but not for validation. All features assembled during model training and testing were assessed Feature importance Was evaluated for both train and test sets using permutation feature importance, which analyzes how model performance is influenced by randomly shuffling the association between particular features and the outcome of interest. In order to reduce the final feature set we used the permutation feature importance computed on the test set. The features with permutation feature importance equal to or below zero were eliminated from further analysis, reducing the PSC feature sets sizes to 27 for RF and 14 for XGB, while the nPSC XGB was reduced to 14 features and the nPSC RF set remained unchanged.

While we initially used only a single representative unitig from a covarying set of identically or nearly identically distributed unitigs, we used all unitigs in the covarying sets for the functional annotation. When we included all unitigs from each covarying set, our best performing PSC models expanded to 111 and 176 features for RF and XGB models respectively, while the best performing nPSC models expanded to 83 and 444 features for RF and XGB respectively (S6 Fig). We then mapped these expanded unitig sets to the Pa genome and recorded the corresponding coding sequence or upstream and downstream coding sequences when the unitig mapped to a non-coding region. The number of features in coding and non-coding regions showed no significant difference between PSC and nPSC (Chi-squared test, p-value = 1.0) (S7 Fig). The final list of genes associated to AET success/failure was of 95 for RF PSC, 102 for XGB PSC, 322 for XGB nPSC, and 68 for RF nPSC. Several unitigs mapped to the same genes (S2 Table). Here we focus on the genes encountered using the PSC RF model since was the one with the highest performance scores.

We classified the genes into eight functional categories: virulence, biofilm, antimicrobial resistance, CF-associated (genes whose variation has been previously reported to impact Pa adaptation to the CF lung), metabolism, transcriptional regulation, and mobile genetic elements. 28% of mapped genes were identified as virulence factors, while 28% of the genes were annotated as hypothetical proteins with unknown function (S3 Table).

The top ranked covarying unitig set for the RF PSC pipeline was composed of one unitig that mapped to PopB, a translocation protein that interacts with the T3SS. The second group included only one feature that mapped to the transcriptional regulator soxS. Other featured genes found include *pelB*, *bapA*, *ecpE/cup*, and *fliK*, which are genes frequently associated with pathogenicity and CF persistent isolates. PelB is involved in pellicle polysaccharide synthesis, which is an important component of the biofilm structure and mediates tolerance to aminoglycosides [70,71]. BapA is involved in adhesion and biofilm formation [72]. FliK is the flagellar hook length control protein associated with swarming motility abilities [73]. And EcpE/Cup is a fimbrial chaperone in *E*. *coli* with a role in early stages of biofilm formation and host cell recognition [74].

We also found virulence-associated genes such as *fimA*, *algI*, *clpP*, *nhaP2 popB*, *hxuA* [75,76]; quorum sensing *qseC*, *phnB*, *sasA* [77,78]; iron scavenging *fhuA*, *pchE* [79–81], as well *purK* and *topA*, which are associated with adaptation to the CF lung [82,83]. One of the selected features that showed a high association to the persistent samples was a non-coding variant between *motY/pyrC*, which is an intergenic region associated with adaptation to the CF lung [84]. Also highly associated to persistent samples were variations in *rhsC*, *msrB*, which are related to oxidative stress, and *ompU*, which is associated with cell permeability. One feature set was selected with three models (PSC XGB and RF, and nPSC RF) and showed a strong association with persistent samples. This set of 24 covarying unitigs mapped to *comEC*, *argJ*, *phbB*, *pcaK*, *preA*, *algI*, and hypothetical proteins, with most mapped to *comEC*, which is a gene necessary for natural transformation in many bacterial taxa [85].

The groups of covarying features suggest target genes and variants for further exploration. However, since these features are highly correlated, not all of them may be causative of treatment failure but could be linked with casual variants.

## Discussion

Pa chronic infections in CF pediatric patients have been extensively studied. Pa persistent isolates have been shown to have phenotypic and genotypic characteristics associated with adaptation to the CF lung. Less has been explored on the genetic background of new-onset Pa isolates, usually environmentally acquired, that lead to persistence. In this study, we developed random forest predictive models using a nested cross validation (NCV) design and population structure control (PSC) to predict the outcome of the AET for new-onset Pa infections in children with CF. Our sample consisted of Pa isolates recovered prior to AET, as well as a small set of isolates recovered after AET failure.

The use of machine learning to predict traits or outcomes of interest based on genomic data is becoming an increasingly popular and important tool in microbial genomics. While these approaches hold great promise, most studies must find ways to mitigate several intrinsic data characteristics that can negatively impact machine learning model performance. One of these characteristics is the imbalanced sample composition (i.e., many more samples from one outcome or trait class than the other), that if not addressed properly, leads to the under-learning of the minority class. In the worst case, a model can be trained that by chance does not include any representative from the minority class. To overcome this issue, we fixed the class proportions based on their observed values in the total dataset. This ensured that the minority (AET failure) class was adequately represented in both the train and test sets. We also selected the best performing models based on the minority class metrics such as precision and recall.

A frequent problem encountered in statistical genomics is high dimensionality data (i.e., many variables or features) with a small sample size, or the so-called large-p, small-n problem [86]. This problem is particularly challenging when it is difficult or expensive to collect samples, as is commonly the case in clinical research. This spare dataset problem can lead to ascertainment bias and over-optimistic model performance estimates [87]. We addressed this concern by performing a rarefaction analysis on the unitig to identify the sampling depth needed to interrogate our sequence diversity (S8 Fig). To avoid overfitting, we used NCV and a train/test split approach, this combination produces robust and unbiased estimations [25,87]. Other ways to deal with high dimensionality data include dimensionality reduction and feature selection. We performed dimensionality reduction with MCA and applied filters and wrapping methods on unitig patterns. The former MCA showed good performance but had poor resolution and generates features that are difficult to interpret. Ultimately, *we used* filtering methods and recursive feature elimination to identify those features that had the greatest impact on model performance. These approaches allowed us to greatly reduce the very large number of genomic unitig variants used as input features while maintaining predictive power and increasing interpretability (PSC RF = 30, nPSC RF = 14, PSC XGB = 24, nPSC XGB = 15).

Despite focusing on the most important features, individual unitigs showed only weak correlation with the AET outcome, suggesting that AET failure during new-onset Pa infections is a complex polygenic trait. The annotation of high performing unitigs points to genes with roles in adhesion, motility, and biofilm formation, all of which are known to contribute to reduced effectiveness of antibiotics. The functional redundancy observed in the featured genes implies that there are multiple mechanisms that can increase the likelihood of AET failure persistence. We showed that the antibiotic resistance profiles of the isolates recovered from successful and failed AET infections were significantly different only for ciprofloxacin, gentamicin, and imipenem, although post-failure isolates, recovered around a month after AET treatment, do show increased MIC levels for all antibiotics tested except colistin. This increase in antibiotic resistance could be due to reduced permeability or increased expression of efflux pumps as suggested by the functional annotation of unitigs associated to post failure samples.

Another important statistical genomics consideration, particularly when dealing with microbial samples, is population structure, which is non-independence of the samples caused by shared evolutionary history. We addressed this issue by blocking by BAPS groups as a PSC, which involved assigning all the strains within each BAPS group to either the train or the test datasets during the NCV outloop and feature selection process. Since BAPS groups generally correspond to phylogenetic clades (i.e., monophyletic lineages of strains), this approach effectively examined how robustly the model performed when working with previously unseen lineages of strains. Not surprisingly, models with and without PSC yield different results and selected different features. While the nPSC models generally had better performance, the prediction error distribution across the phylogeny shows that the PSC models identified features that were less dependent on the evolutionary relationships among strains, resulting in a much more generalizable model, and making these features strong candidates for future studies of treatment failure.

Despite the efforts placed into producing models that have high performance and generalization power, they are still based on one-hot encoded sequence diversity, which means that variation found in the population but not in our sample could not be modeled. This is where the mapping and annotation of the selected features (i.e., determining what genes carry the variant feature of interest) becomes of great importance. Even though unseen variation cannot be accounted for, the approach used allows us to identify genes and pathways putatively involved in AET failure and the establishment of chronic infections. It was gratifying to see that many of our PSC selected features mapped to known virulence-associated genes, such as biofilms, iron metabolism and scavenging, motility, and quorum sensing.

In summary, our approach effectively predicted AET failure among new-onset Pa infection in children with CF using Pa genomic sequences. We also showed that including controls for population structure in the analysis is necessary for generalizability and biological interpretation. The power of this approach is made even more evident given the fact that no information on the host factors, comorbidities, or other clinical or environmental data were included in this analysis. These powerful methods provide new avenues for the analysis of high dimensional genomic data and are likely to play a prominent role in predicting complex phenotypes that are underpinned by many polymorphic genes interacting with each other and a suite of environmental and host factors in unpredictable ways.

## Methods

### Sample collection & ethics

The study cohort consisted of 70 cystic fibrosis pediatric patients from The Hospital for Sick Children, Toronto, Canada, with at least one new-onset Pa infection registered between 2011–2016. Formal consent was obtained for the study and approved by the Research Ethics Board of the Hospital for Sick Children (REB#1000061322). Written informed consent was obtained from the parent/guardian of each participant under 18 years of age. The average age of the patients is 9.7 years (with a standard deviation of 3.5) and 51% of them are female. Further information on the cohort can be found in [59]. Pa isolates were recovered from sputum samples collected before antibiotic treatment and sequenced. If colony morphology variation was observed, different isolates were sequenced to represent the diversity. New-onset Pa infection was defined as first lifetime acquired infection or having a Pa-positive sputum culture after having at least three Pa-negative sputum cultures within the prior 12 months. Patients are typically monitored for PA infections every three months with routine culturing of respiratory tract specimens. AET failure (persistent isolates) was defined as having a positive sputum culture for Pa on the culture done 1 week after completion of AET. Eradicated cases were defined as having a negative sputum culture for Pa in the same time frame. Isolates retrieved from sputum obtained after treatment failure were also sequenced for 10 patients (post-failure samples) to confirm AET outcome assignation. The antibiotic treatment consisted of a multi-step protocol of inhaled tobramycin followed by intravenous tobramycin and ceftazidime detailed in [15].

### Antimicrobial susceptibility testing

All Pa isolates were screened for antimicrobial susceptibility by the broth micro-dilution method in accordance with Clinical and Laboratory Standards Institute (CLSI) procedures [88]. Susceptibility profiles were determined for β-lactams (aztreonam, ceftazidime, cefepime, meropenem, imipenem), fluoroquinolones (ciprofloxacin, levofloxacin), aminoglycosides (amikacin, tobramycin, gentamicin), β-lactams/β-lactamase inhibitor (piperacillin/tazobactam) and colistin. Isolates were grown in Mueller-Hinton II broth overnight at 35°C in a two-fold dilution series of each antibiotic. Results are reported as minimum inhibitory concentration (MIC), and resistant, susceptible or intermediate phenotypes were assigned according to CLSI guidelines.

### Genome analysis

All sequencing was performed on the Illumina NextSeq instrument at the University of Toronto Centre for the Analysis of Genome Evolution and Function (CAGEF). WGS raw data was trimmed using Trimmomatic [89] (LEADING:3 TRAILING:3 SLIDINGWINDOW:4:15 MINLEN:80) and assembled with Spades v3.14.1 (—careful -k 21,33,55,77,83,91,101,113,121,127 –mismatch-correction) [90]. Annotation was performed with Prokka [91]. Reference genomes were obtained from RefSeq database (PAO1: GCF_000006765.1, PA14: GCF_000404265.1, PA7: GCF_000017205.1) and re-annotated with Prokka. Pangenome analysis were performed using Roary (-I 95 -e–-mafft) [92]. RaxML [93] was used for core genome based phylogenetic reconstructions with parameters (-m GTRGAMMA -p 12345 -# 20). Bayesian analysis of population structure were performed using hierBAPS in R with default parameters [94]. Core genome distances were estimated using MASH [95], and accessory genome distances were estimated using Bray-Curtis dissimilarity index from the gene presence absence matrix obtained with Roary [92].

### Machine learning and model evaluation

#### Encoding genomic variation

We used unitig-counter [63] to create unitigs from the genome assemblies. Unitigs are short sequences of different length that can represent different forms of genetic variation such as SNPs and indels and can dispense with the use of a reference genome. Each genome is represented by a vector of presence/absence of each unitig sequence. A presence-absence matrix for 494 genomes with a total of 542,296 unique unitig patterns was generated. Low and high frequency patterns (<5%, >90%) were removed, a total of 425,005 features remained for further analysis.

#### Feature selection, model independent selection

Filters were applied on train data only. We calculated Chi-square statistic between each feature and the target and select the desired number of features with best Chi-square scores (p-value < 0.01). Then, we created a correlation matrix with Pearson method and dropped highly correlated features (Pearson correlation coefficient > 0.7) leaving 4800 unique unitig patterns for training.

#### Feature selection, model dependent selection

We used recursive feature elimination (RFE) with a random forest predictor to select features that are more relevant in predicting the target variable. We compared independent runs with different numbers of features to select (RFE: n to select = 100,50,30,10) in both PSC and nPSC pipelines.

#### Model design and evaluation

Random forest implementation of scikit-learn (v0.24.1) package in python (v3.6.8) with parameters adjusted during tuning via cross validation was used to build the predictors [96]. We compared two model designs, one consisted of nested cross validation (NCV) with blocking by BAPS groups to control the population structure, and one without blocking. Blocking was applied during the outer loop of the nested cross validation, restricting the use of certain BAPS subpopulations to either the test (15% of the data) or the train (85% of the data) set. We used *GroupShuffleSplit* function to apply blocking taking class proportion into account. Train/test outer split of the models with no PSC were based on class proportion information only (*StratifiedShuffleSplit*).

In every case, during the inner loop the hyperparamter search was performed using the function *GridSearchCV* with cv = 3. The hyperparameters combination tested was: n_estimators: 50, 100, 200, class_weight: None, balanced, max_depth: 3, 4, 5, 6, 7, 8. With the best hyperparameter combination chosen, the RFE step is run, where features are selected. The selected features from each outer loop that showed train performance above 0.7 AUC, were combined to fit new predictors. Random Forest and Extreme gradient boosting models were fitted with the selected features.

#### Performance metrics

We used the area under the ROC curve (AUC) for assessing overall performance on training and test sets or training and validation sets. ROC is a probability curve and AUC represents the degree of separability, how much the model is capable of distinguishing between classes. A high AUC indicates low prediction error. Precision, recall and F1-score were estimated for both classes using the *classification_report* function from sickit-learn [96]. Precision measures of how many of the positive predictions made are correct (true positives), recall measures how many positive cases were detected over all the positive cases (sensitivity). F1-score is a single metric that weights precision and recall. The metric used for evaluating feature importance was Gini importance or mean decrease impurity. Permutation importance was also estimated using *permutation_importance* function from scikit-learn (permutations = 100).

#### Feature annotation

The one-hot encoded input features represent presence/absence of certain unitig sequences in the genomes. In order to evaluate function and localization of the relevant variation we mapped the unitig sequences to the genomes. The unitigs patterns were mapped using a python script from pyseer [97]. When unitigs mapped onto noncoding regions, the genes up and down stream were identified. All genomes in our database were used as references so that all the patterns could be annotated, and prokka annotations was improved using MicrobeAnnotator [98], the Virulence Factor Database [99], and the KEGG database [100].

## Supporting information

S1 Table Samples used in the study. (XLSX)

S2 Table Features selected with all pipelines. (XLSX)

S3 Table Genes selected with the best performing model, PSC RF, with detailed information on gene function. (XLSX)

S1 Fig Samples reassignment and phylogenetic diversity. (A) Core genome, midpoint-rooted phylogeny of the 494 Pa strains. AET outcome is represented by the color coding of the dashed lines leading from the terminal nodes to the metadata rows, with blue showing AET success (i.e., eradication), red showing AET failure, and orange showing a post-failure isolate. The annotation bar indicates the patients that showed within infection variation and therefore post-treatment isolates were sequenced. Pre- and post-treatment samples share a common ancestor, except for one post failure sample, corresponding to patient SK069. (B) Accessory genome distances between pre- and post-treatment samples. Comparisons were made within patients that showed genetic variation. Bray-Curtis distances were estimated from the pangenome presence-absence gene matrix. Strain designations enclosed red or orange indicate failure and post-failure isolates, while those enclosed in black indicate isolates whose phenotype were modified from failure to eradication due to accessory genome distance. (C) Proportion of samples in each class (eradication and failure) before (left panel) and after (right panel) the samples reassignment and post failure inclusion. (D) Unrooted phylogeny created using the reference genomes PAO1, PA7, LESB58, and PA14. None of our samples were found related to the reference PA7, therefore we removed PA7 to improve the visualization. (E) The phylogeny without PA7 and midpoint rooted. The references, PAO1, PA14 and LESB58 are highlighted.
(TIF)

S2 Fig Training/test splits generated using PSC and without PSC during the outer loop of NCV. (A) Train/test splits with population structure control (PSC). The top panel displays how treatment outcome (class) proportions are maintained across all splits. The panel below illustrates the same train/test splits but color-coded based on BAPS subpopulations. For the PSC models, specific BAPS group can only be in either the train or test, but not in both, conditioning also the size of the splits. (B) Train/test splits without population structure control (nPSC). The top panel displays how treatment outcome (classes) proportions are maintained across all splits. The panel below illustrates the same train/test splits but color-coded based on BAPS subpopulations, noting that BAPS groups can be present in both train and test splits.
(TIF)

S3 Fig Random Forest design and performance overview. (A) Decision trees use tree representations to solve problems, in which leaves represent class labels and internal nodes represent attributes. A random forest is an ensemble of many individual decision trees, each tree’s classification is combined into a final classification through a "majority vote" mechanism. (B) A schematic of the decision boundary (partition of the feature space) showing correct and incorrect samples predictions. (C) Model performance. In the confusion matrix, the rows represent the true labels, and the columns represent the predicted labels. Diagonal values represent the number of times the predicted label matches the true label. Observations in the other cells were mislabeled by the classifier. From the confusion matrix, precision, recall, and F1 score can be derived. A ROC curve (receiver operating characteristic curve) is a graph showing the performance of a classification model at all classification thresholds. This curve plots two parameters, the true and false positive rates. The area under the ROC curve (AUC) provides an aggregate measure of performance across all possible classification thresholds. AUC indicates how well the model can distinguish between classes. The higher the AUC, smaller the prediction error.
(TIF)

S4 Fig Predictive power of randomly selected features. We fit 500 models with 500 subsets of 25 randomly selected features from the uncorrelated 4800 features set. Test (top panel) and train (bottom) AUC values for the 500 models are shown. In green the AUC values for test and train obtained with the best performing models of the no population structure control (nPSC) pipeline, and in red the test and train values of the best performing models with population structure control (PSC). Randomly selected features can show high accuracy most likely due to the correlation of AET outcome and the phylogeny.
(TIF)

S5 Fig Association of selected features patterns with the antibiotic treatment outcome distribution. (A) For each feature selected with PSC random forest during the population structure control pipeline (RF PSC) the comparison of the distribution of the unitig presence/absence pattern in both the eradication and failure groups. (B) The distribution of the unitig presence/absence pattern is now distributed in three groups, eradication, failure and post-failure, to assess the impact of the latter for each independent feature.
(TIFF)

S6 Fig Permutation Feature Importance and covarying sets sizes. (A) Covarying sets of features selected with the Random Forest no population structure control (nPSC) pipeline. (B) Covarying sets of features selected with the Random Forest population structure control (PSC) pipeline. (C) Covarying sets of features selected with the Extreme Gradient Boosting with nPSC. (D) Covarying sets of features selected with the Extreme Gradient Boosting with PSC.
(TIFF)

S7 Fig Feature annotation summary. (A) Proportion of core, soft core, shell, and cloud genes selected with each pipeline. (B) Proportion of coding and non-coding regions mapped with each pipeline.
(TIF)

S8 Fig Unitig Pattern Rarefaction Curve. (TIF)
