# Supplementary figures and images for: Predictive modeling of antibiotic eradication therapy success for new-onset Pseudomonas aeruginosa pulmonary infections in children with cystic fibrosis
Source: PLoS Comput Biol. 2023 Sep 6;19(9):e1011424. doi: 10.1371/journal.pcbi.1011424 (PMC10506723; doi:10.1371/journal.pcbi.1011424)

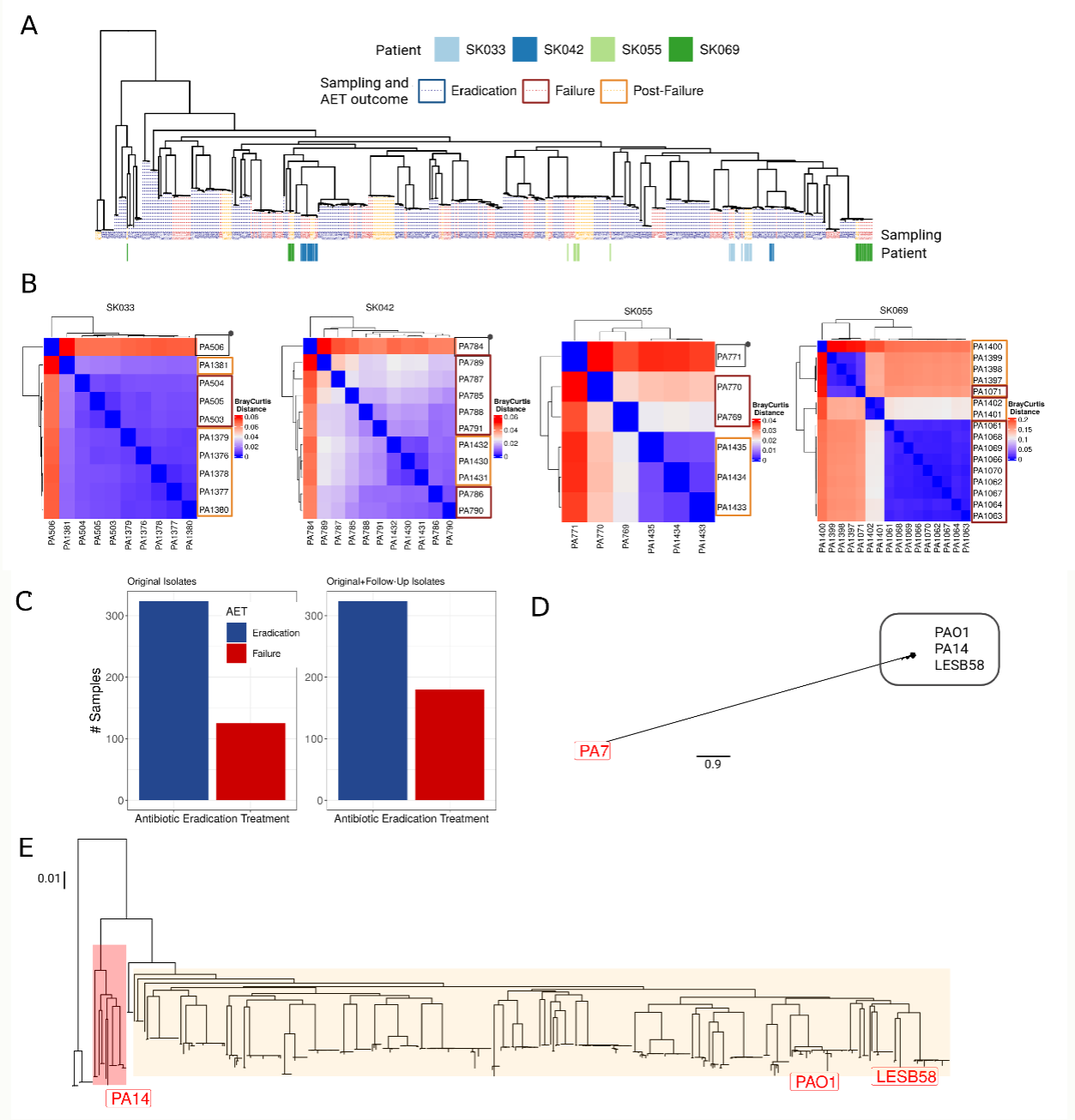

Supplement: S1 Fig — (A) Core genome, midpoint-rooted phylogeny of the 494 Pa strains. AET outcome is represented by the color coding of the dashed lines leading from the terminal nodes to the metadata rows, with blue showing AET success (i.e., eradication), red showing AET failure, and orange showing a post-failure isolate. The annotation bar indicates the patients that showed within infection variation and therefore post-treatment isolates were sequenced. Pre- and post-treatment samples share a common ancestor, except for one post failure sample, corresponding to patient SK069. (B) Accessory genome distances between pre- and post-treatment samples. Comparisons were made within patients that showed genetic variation. Bray-Curtis distances were estimated from the pangenome presence-absence gene matrix. Strain designations enclosed red or orange indicate failure and post-failure isolates, while those enclosed in black indicate isolates whose phenotype were modified from failure to eradication due to accessory genome distance. (C) Proportion of samples in each class (eradication and failure) before (left panel) and after (right panel) the samples reassignment and post failure inclusion. (D) Unrooted phylogeny created using the reference genomes PAO1, PA7, LESB58, and PA14. None of our samples were found related to the reference PA7, therefore we removed PA7 to improve the visualization. (E) The phylogeny without PA7 and midpoint rooted. The references, PAO1, PA14 and LESB58 are highlighted. (TIF) [file pcbi.1011424.s004.tif]

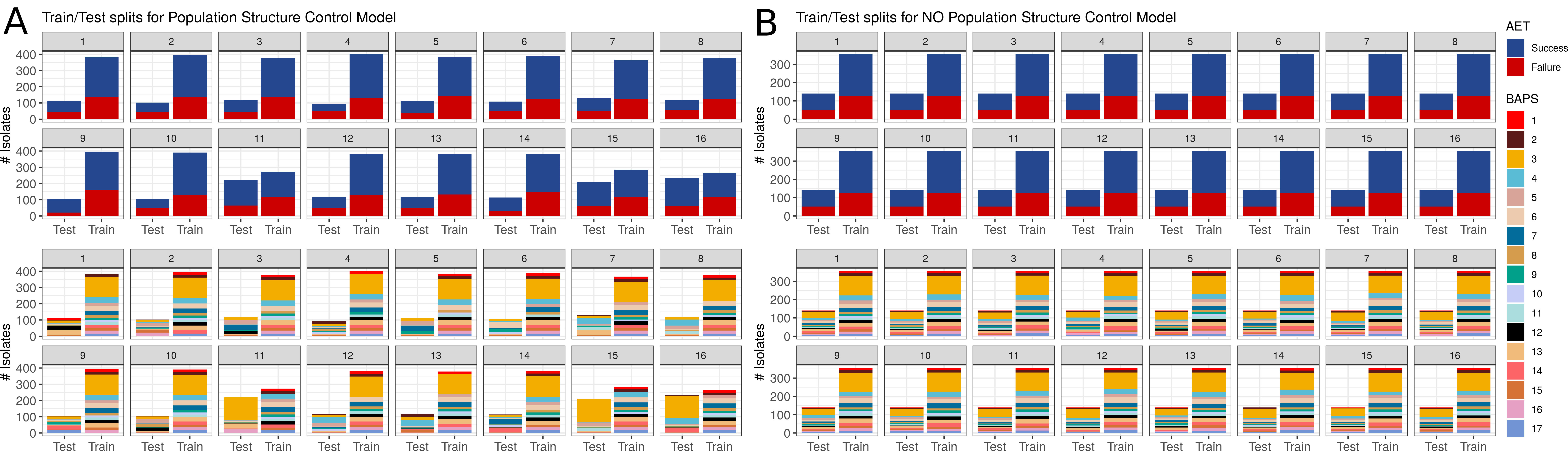

Supplement: S2 Fig — (A) Train/test splits with population structure control (PSC). The top panel displays how treatment outcome (class) proportions are maintained across all splits. The panel below illustrates the same train/test splits but color-coded based on BAPS subpopulations. For the PSC models, specific BAPS group can only be in either the train or test, but not in both, conditioning also the size of the splits. (B) Train/test splits without population structure control (nPSC). The top panel displays how treatment outcome (classes) proportions are maintained across all splits. The panel below illustrates the same train/test splits but color-coded based on BAPS subpopulations, noting that BAPS groups can be present in both train and test splits. (TIF) [file pcbi.1011424.s005.tif]

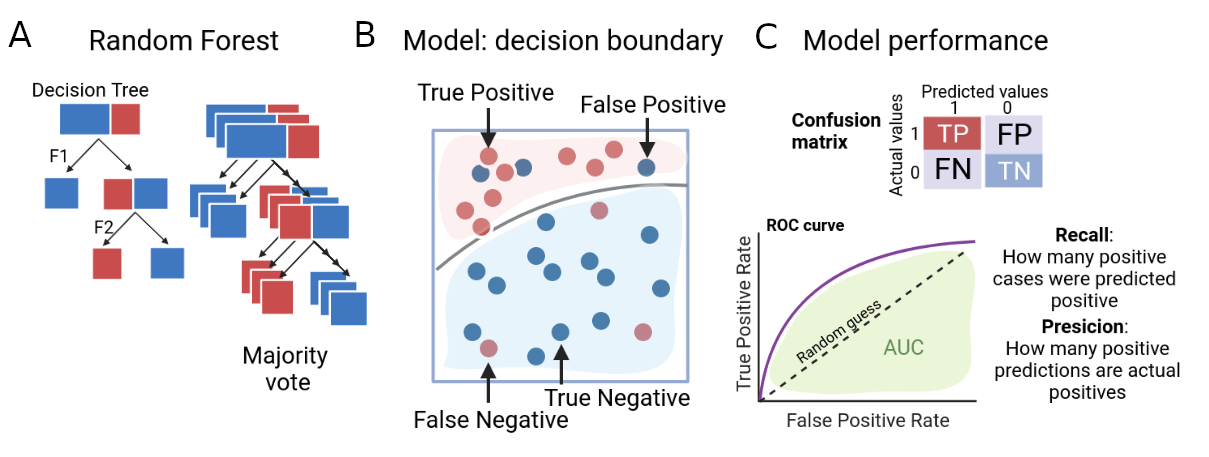

Supplement: S3 Fig — (A) Decision trees use tree representations to solve problems, in which leaves represent class labels and internal nodes represent attributes. A random forest is an ensemble of many individual decision trees, each tree’s classification is combined into a final classification through a "majority vote" mechanism. (B) A schematic of the decision boundary (partition of the feature space) showing correct and incorrect samples predictions. (C) Model performance. In the confusion matrix, the rows represent the true labels, and the columns represent the predicted labels. Diagonal values represent the number of times the predicted label matches the true label. Observations in the other cells were mislabeled by the classifier. From the confusion matrix, precision, recall, and F1 score can be derived. A ROC curve (receiver operating characteristic curve) is a graph showing the performance of a classification model at all classification thresholds. This curve plots two parameters, the true and false positive rates. The area under the ROC curve (AUC) provides an aggregate measure of performance across all possible classification thresholds. AUC indicates how well the model can distinguish between classes. The higher the AUC, smaller the prediction error. (TIF) [file pcbi.1011424.s006.tif]

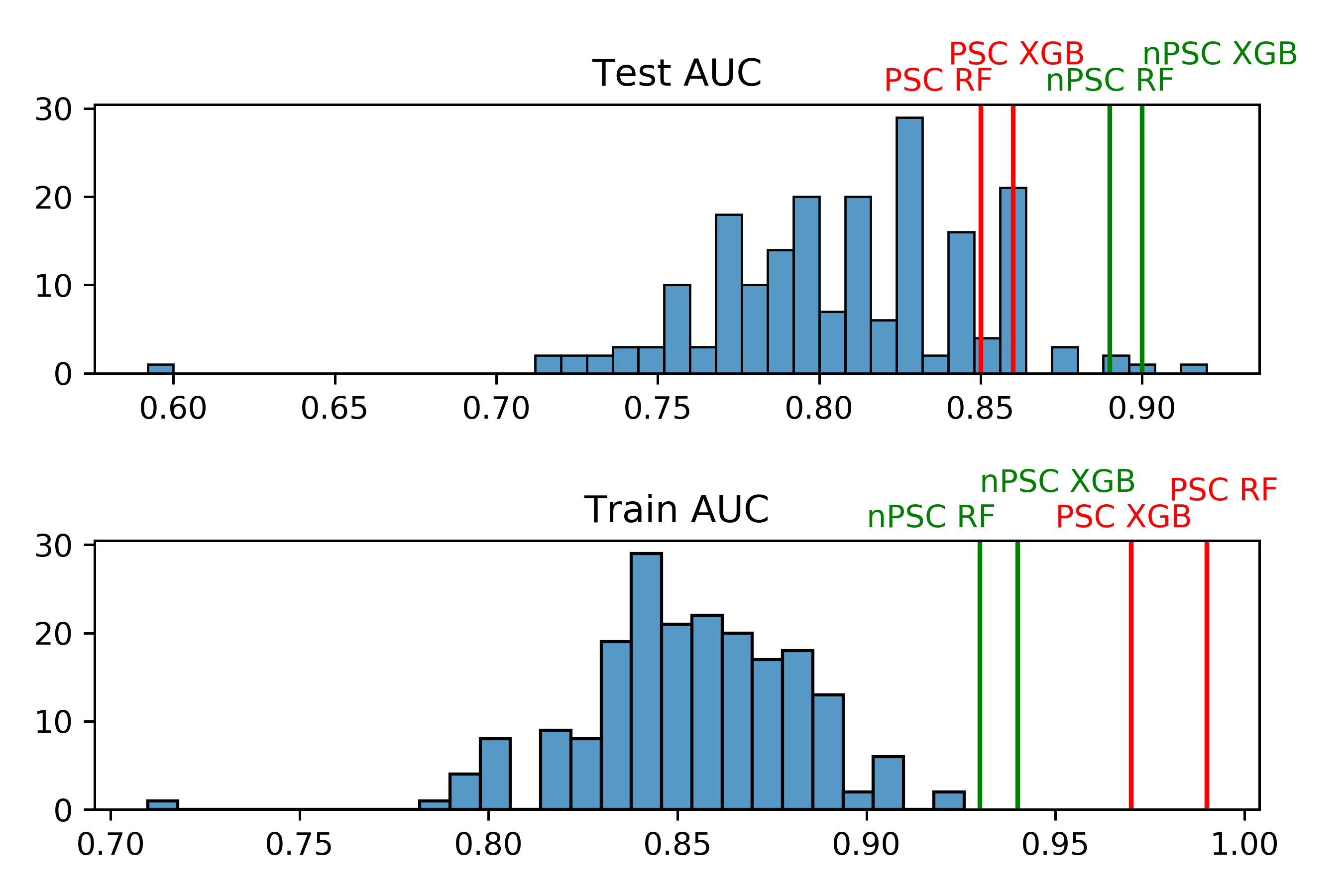

Supplement: S4 Fig — We fit 500 models with 500 subsets of 25 randomly selected features from the uncorrelated 4800 features set. Test (top panel) and train (bottom) AUC values for the 500 models are shown. In green the AUC values for test and train obtained with the best performing models of the no population structure control (nPSC) pipeline, and in red the test and train values of the best performing models with population structure control (PSC). Randomly selected features can show high accuracy most likely due to the correlation of AET outcome and the phylogeny. (TIF) [file pcbi.1011424.s007.tif]

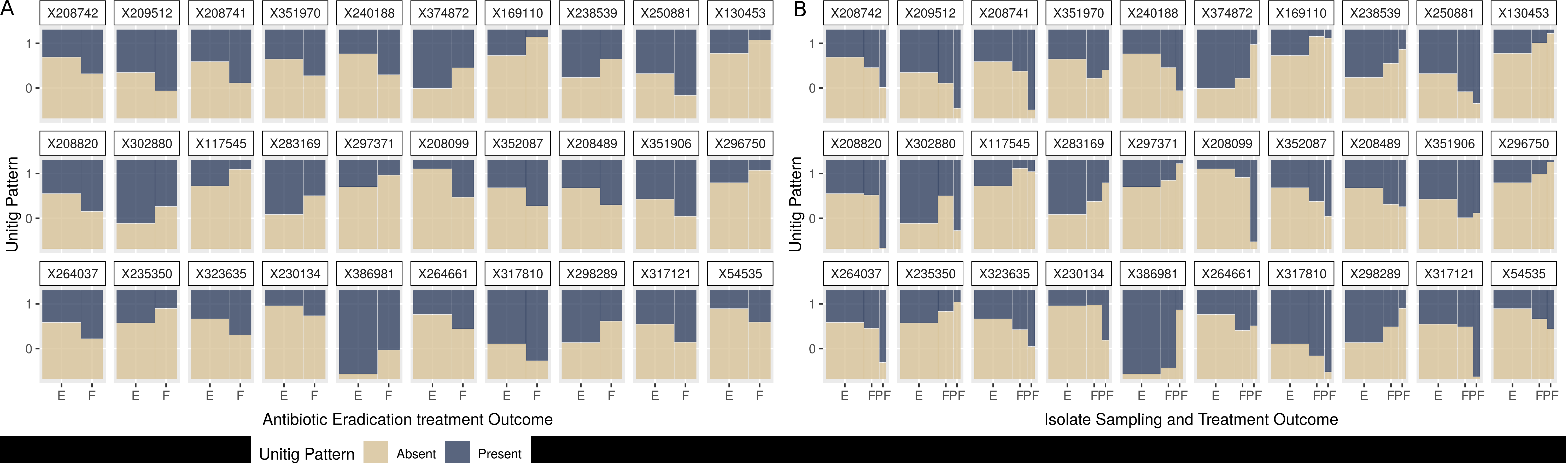

Supplement: S5 Fig — (A) For each feature selected with PSC random forest during the population structure control pipeline (RF PSC) the comparison of the distribution of the unitig presence/absence pattern in both the eradication and failure groups. (B) The distribution of the unitig presence/absence pattern is now distributed in three groups, eradication, failure and post-failure, to assess the impact of the latter for each independent feature. (TIFF) [file pcbi.1011424.s008.tiff]

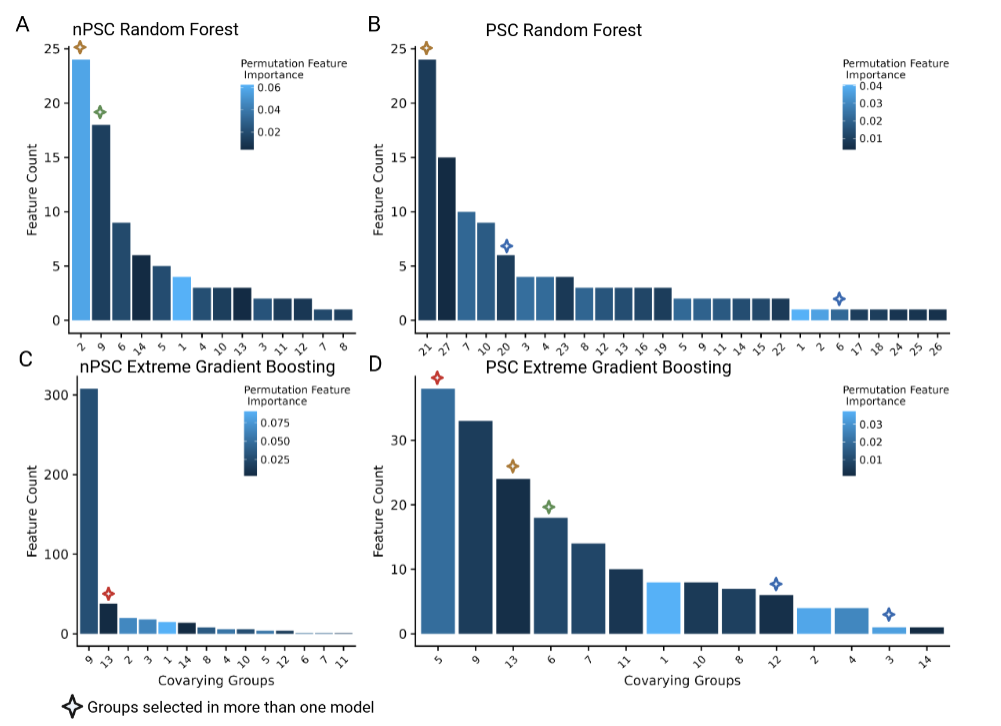

Supplement: S6 Fig — (A) Covarying sets of features selected with the Random Forest no population structure control (nPSC) pipeline. (B) Covarying sets of features selected with the Random Forest population structure control (PSC) pipeline. (C) Covarying sets of features selected with the Extreme Gradient Boosting with nPSC. (D) Covarying sets of features selected with the Extreme Gradient Boosting with PSC. (TIFF) [file pcbi.1011424.s009.tiff]

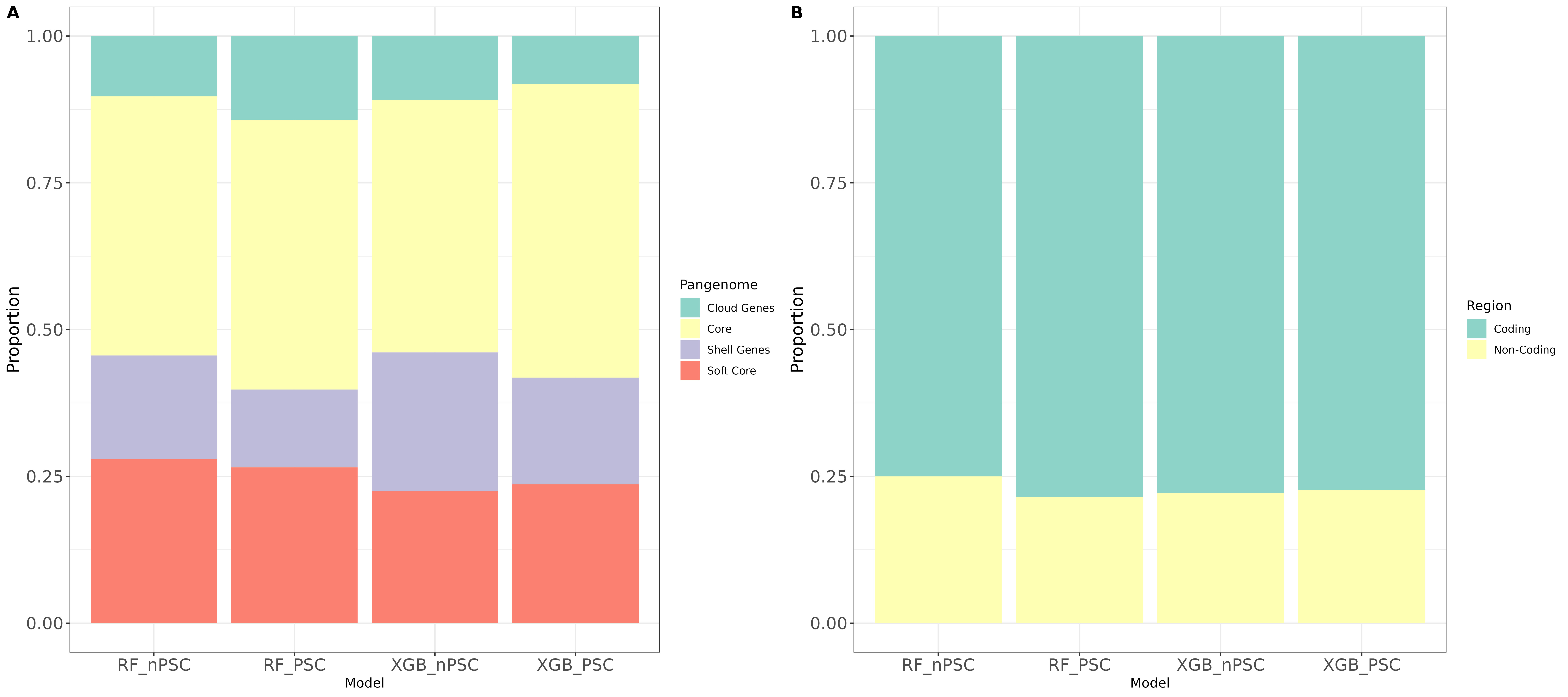

Supplement: S7 Fig — (A) Proportion of core, soft core, shell, and cloud genes selected with each pipeline. (B) Proportion of coding and non-coding regions mapped with each pipeline. (TIF) [file pcbi.1011424.s010.tif]

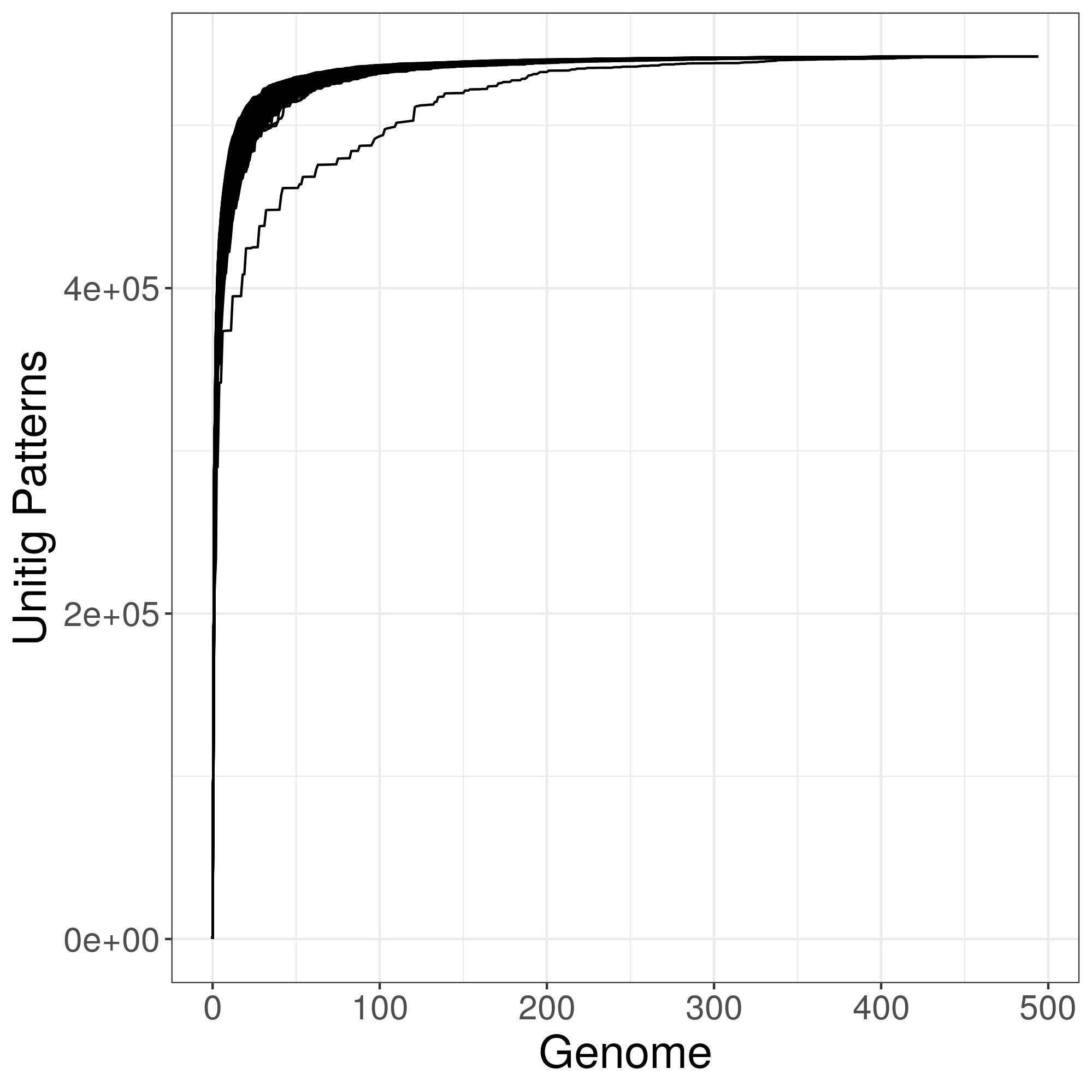

Supplement: S8 Fig — (TIF) [file pcbi.1011424.s011.tif]
